# The effect of ovulation synchronization with OvuGel on ovarian follicles and fertility responses to a single fixed time insemination in different parities and seasons

**DOI:** 10.1093/tas/txaf052

**Published:** 2025-04-18

**Authors:** Lidia S Arend, Amanda M Minton, Clint R Schwab, Caleb M Shull, Stephen G Buysse, Mike E Johnston, Catherine M Roaten, Christopher L Anderson, Stephen K Webel, Robert V Knox

**Affiliations:** Department of Animal Science, University of Illinois, Champaign-Urbana, IL 61801, USA; AcuFast, Breese, IL 62230, USA; AcuFast, Breese, IL 62230, USA; The Maschhoffs, LLC, Carlyle, IL 62231, USA; United Animal Health, Sheridan, IN 46069, USA; United Animal Health, Sheridan, IN 46069, USA; United Animal Health, Sheridan, IN 46069, USA; United Animal Health, Sheridan, IN 46069, USA; United Animal Health, Sheridan, IN 46069, USA; Department of Animal Science, University of Illinois, Champaign-Urbana, IL 61801, USA

**Keywords:** OvuGel, ovulation, parity, single insemination, sows, season

## Abstract

Increasing use of semen from superior sires can accelerate genetic improvement when using a single fixed-time AI (SFTAI) to reduce the number of sperm required to produce a litter. The objectives of this study were to: 1) Assess whether follicle development, ovulation, and insemination was altered by parity and season in OvuGel and Controls; 2) Determine the impact of Day of OvuGel administration and estrus in Controls; and 3) Evaluate OvuGel and Control interactions with parity and season on farrowing rate and litter size. The experiment was performed in replicates in summer and fall with sows assigned from Monday and Thursday wean groups by parity to OvuGel or Control after weaning (Day 0). Sows received OvuGel (n = 1,636) on Days 3, 4 or 5 based on proestrus or estrus symptoms and then a SFTAI 24 h later. Controls with a wean to estrus interval of 3 to 6 d (n = 1,676) received an AI on each day standing. Ovaries of a sub-population of the Thursday weaned sows (n = 445) were scanned by ultrasound. The number (17.5) and size (7.0 mm) of ovulatory follicles on Day 4 did not differ between treatments, but P1 and P2 sows had smaller follicles and sows weaned in Sep-Oct had fewer and smaller sized follicles (*P* ≤ 0.05). OvuGel increased (*P* = 0.004) ovulation by Day 6 (92.2%) compared to Control (79.4%). Sows with large follicles on Day 4 were more likely to ovulate (87.8%, *P* < 0.0001) by Day 6 than those with medium (62.1%). Although there was no interaction with parity, ovulation was lower in the Control for parities 1, 2, and ≥ 7. OvuGel treatment on Days 3 or 5 reduced (*P* = 0.0003) farrowing rate (FR) by 7 to 11% and were excluded from further analyses. Day 4 OvuGel treatment had no effect on FR (86.7 vs. 87.4%) or total born (TB) compared to Control (13.2 vs. 13.4), respectively. Interactions were due to OvuGel improving FR in P1 sows (87.9 vs. 76.5%) and for sows weaned in Jul-Aug (89.2 vs. 83.4%) compared to Control, respectively. There was an effect of day of weaning that was linked to semen storage (*P* = 0.02) indicating 4 to 5 d (84.5%) reduced FR compared to ≤ 3 d (91.3%). The effect was evident with the SFTAI but not Control and was thought to originate with changes in fertility with additional days of handling and storage in summer. The results indicate OvuGel treatment on Day 4 at proestrus or estrus and followed by a SFTAI has great potential to mimic the fertility performance of conventionally inseminated sows.

## Teaser

OvuGel increased the proportion of sows ovulating within 6 d after weaning and when given on Day 4 at proestrus or estrus and followed by a single fixed time artificial insemination (n = 1,636), farrowing rate (87.5%) and total born (13.3) were similar to Controls. Further, OvuGel increased fertility for parity 1 sows and for females weaned in Jul-Aug compared to Control.

## INTRODUCTION

There could be significant genetic and fertility improvements when using advanced technologies such as single fixed-time artificial insemination (SFTAI) (Kraeling and Webel, 2015). One product approved for use in swine, OvuGel, and other similar products rely on administration of GnRH or an agonist at a defined time post-weaning followed by a SFTAI ~24 h later. The success of the procedure relies on sows having preovulatory follicles at the time of induction. If the sows are fertile, this method can induce a high proportion of females (80% to 90%) to ovulate within 24 h of each other (Knox et al., 2018), which would facilitate a single insemination to occur within the optimal window before ovulation. As a method to only include fertile sows, Dillard and Flowers (2020) treated sows in proestrus or estrus on Day 4 after weaning with OvuGel and then inseminated and were able to achieve excellent fertility. Although sows with slower follicular growth and others with delayed estrus after weaning did tend to have lower fertility. It is uncertain, however, if this technology may be affected by parity and season, since both have been associated with reduced follicle development, estrus, and farrowing (Soede et al., 2011; Lopes et al., 2014; De Rensis and Kirkwood, 2016).

At present, estrus remains the key indicator of fertility for pig breeding because there are no practical ways for commercial farms to determine which sows have mature follicles after weaning. Studies have indicated that the fertility of sows subjected to FTAI is influenced by the development of large follicles and the expression of estrus (Martinat-Botté et al., 2010; Knox et al., 2011; Driancourt, 2013; De Rensis and Kirkwood, 2016). Methods to improve the fertility of sows assigned for FTAI have included the use of exogenous gonadotropins or procedures to identify symptoms of proestrus or estrus. While proestrus symptoms would have potential, waiting until estrus for individual sows might not achieve the same degree of ovulation synchronization (Knox et al., 2011). Nevertheless, an advantage to a FTAI program is that breeding does not need to rely only on estrus. In fact, one of the greatest challenges for modern swine breeding systems is detection of estrus (Kemp and Soede, 2012) since the response can sometimes be subjective, and influenced by technician, season, parity, stress, genetics, wean to estrus interval and refractory behavior (Hemsworth and Barnett, 1990; Tummaruk et al., 2000; Belstra et al., 2004; Pedersen, 2007). Poor estrus detection is the most common problem associated with reduced reproductive performance on swine farms (Flowers and Esbenshade, 1993). While the symptoms of estrus may vary, the generally accepted identifier requires the standing reflex in the presence of a boar when using the backpressure test (Kemp et al., 2005; McGlone et al., 2021). Identification of proestrus symptoms is defined by a technician recognizing female interest in the boar, sticky vaginal mucus, reddening of the vulva while allowing for movement and vocalizing, but without a full standing response (Dillard and Flowers, 2020; McGlone et al., 2021).

For success with a SFTAI protocol, sows must be capable of rapidly developing mature follicles after weaning. However, at weaning, the ovarian follicular stage can be heterogeneous and may cause variation in the interval from weaning to estrus (Lucy et al., 2001), duration of estrus, and interval from estrus to ovulation (Kemp and Soede, 1997; Knox et al., 2002). Inseminations that occur within the 24 h period before ovulation are able to fertilize most of the eggs to optimize pregnancy rates and litter size (Soede et al., 1995; Nissen et al., 1997; Rozeboom et al., 1997). However, factors such as parity and season can influence follicle development and interval to estrus after weaning. Parity 1 sows are less likely to display estrus within 7 d after weaning compared to more mature sows, and with these effects exacerbated in summer and fall (Prunier et al., 1996; Peltoniemi and Virolainen, 2006; De Rensis et al., 2017).

Therefore, the objectives of this study were to: 1) Assess whether follicle development, ovulation, and insemination timing was altered by parity and season in OvuGel sows assigned for SFTAI compared to Controls; 2) Determine the impact of Day of OvuGel administration or estrus expression in Controls on fertility; and 3) Evaluate OvuGel and Control interactions with parity and season on farrowing rate and litter size.

## MATERIAL AND METHODS

The use of animals in this experiment was approved by the Institutional Animal Care and Use Committee of the University of Illinois at Urbana-Champaign (#17109).

### Animal Housing and Management

The experiment was performed at a 2,500 sow, breed-to-wean commercial farm in West-Central Illinois. The farm was designed for weekly (Monday and Thursday) weaning groups (80 sows/group). On weaning day (Day 0), sows were moved from the farrowing room to individual gestation stalls located in the breeding and gestation barn, where estrus stimulation and detection were performed once a day from the day of weaning. Females identified in estrus were inseminated and then checked for regular and irregular returns to estrus using a mature boar. Pregnancy diagnosis was performed using transabdominal ultrasound between 28 to 35 d after breeding. After pregnancy was confirmed, sows were moved into groups with 90 females in a pen (1.56 m^2^/sow) that contained an electronic feeding system (Schauer, Austria). The sows remained in the pen until two or three days before the expected farrowing date.

### Experimental Design

This experiment was performed from May to November 2017 in 27 replicates. Each replicate had primiparous and multiparous sows that had their piglets weaned on a Monday or Thursday. The experiment included sows from both wean days with sows (n = 3,312) assigned to treatment (TRT) at weaning. Sows were assigned to OvuGel or Control by parity group (P1, P2, P3 to 6, P ≥ 7), number of pigs weaned (≤ 7, 8 to 14, and ≥ 15), lactation length, and if they had served as a nurse sow. Of sows assigned, approximately 13.4% were P1, 23.7% were P2, 34.4% were P3 to 6, and 28.5% were ≥ P7. Since one of the desired outcomes from a SFTAI program is improved breeding group synchrony, sows assigned to OvuGel were required to show proestrus or estrus On Days 3 to 5 in comparison Control sows that were included if expressing estrus on Days 3 to 6 after weaning. OvuGel (200 µg of triptorelin acetate, United Animal Health, Sheridan, IN, USA) was administered as a 2 mL intravaginal dose deposited 1 to 2 cm posterior to the cervix. A single technician performed the OvuGel application throughout the study and used a multi-dose applicator and a disposable outer sheath for each sow.

The detection of proestrus or estrus was performed once per day starting 24 h after weaning (0900 h) using fence-line exposure to a mature boar while three technicians performed the backpressure test. Boars used for estrous detection were rotated daily, and one of three mature boars was moved into the alleyway in front of the sows in the gestation stalls where females each received 1 to 2 min of fenceline boar exposure. Criteria for identification of proestrus was based on swelling and reddening of the vulva, presence of clear vaginal mucus with a sticky consistency, interest focused toward the boar, but without a standing reflex or erect ears (Dillard and Flowers, 2020). Classification for estrus was defined by females showing the standing reflex with erect ears in response to the backpressure test. Before starting the experiment, the technicians were trained to make the proestrus or estrus decision based on the listed criteria. Females received OvuGel within 1 h after classification of proestrus or estrus on Days 3 to 5 after weaning and were inseminated 24 h after OvuGel administration. Sows assigned to Control were inseminated 45 min after detection of estrus, once on each day standing (maximum 2 to 3 services). Inseminations for both treatments were performed using the post-cervical artificial insemination (PCAI) technique. Two technicians performed inseminations throughout the study using pooled semen doses from 3 to 5 crossbred boars containing two billion motile sperm in 75 mL of a long-term extender. For this study, semen was obtained from a single boar stud with collections on Tuesday and Friday each week and delivery to the sow farm on the same day as collection. Semen was held at the sow farm for 24 h before use, until testing negative for porcine reproductive and respiratory virus.

### Assessment of Follicles and Ovulation

A subpopulation of primiparous (n = 97) and multiparous sows (n = 348) from the Thursday wean group representing both treatments were randomly selected for ultrasound scanning of ovaries to assess follicle size, numbers of follicles, and time of ovulation after weaning. The sows were scanned in 10 replicates every other week during the seasons (months) of May-Jun, Jul-Aug, and Sep-Oct. Transrectal real-time ultrasound was performed using an Aloka 500 V (Hitachi Aloka Medical America, Inc. Wallingford, CT) with a 7.5 MHz linear transducer attached to a PVC stabilizing rod (Knox et al., 2011) with continuous digital recording for playback at a later time to determine or confirm follicle measures. The scans were performed only during Days 4 to 6 at eight-hour intervals, with up to eight scans possible per female. Time of ovulation was defined as the time when ≤ 3 large follicles (≥ 6.5 to 12.9 mm) remained in total from both the right and left ovaries in conjunction with a noticeable (> 50%) reduction in the number of large follicles compared to the previous scan. The scanning of ovaries also identified small (≤ 3.0 mm) and medium (> 3.0 and < 6.5 mm) size follicles as well as follicle cysts (≥ 12.5 mm). Follicle size was measured from 3 to 6 of the largest visible follicles from recordings of the right and left ovaries when possible and used the 3 largest for analysis. The approach for follicle classification and measure has been previously reported and represents the populations described for the pig relevant to ovulation (Knox, 2022). For total follicle counts, observations from both ovaries were required for an individual sow.

### Statistical Analysis

Data were analyzed using ANOVA procedures in SAS (SAS Institute Inc., Cary, NC) and evaluated for normality of residuals (normal distribution) and homogeneity of variance to meet the assumptions for ANOVA using the UNIVARIATE procedure. All models included the main effects of TRT (Control and OvuGel), parity class (P1, P2, P3 to 6, P ≥ 7), season class (early summer, May-Jun (weeks 20 to 26); late summer, Jul-Aug (weeks 27 to 35); early fall, Sep-Oct (weeks 36 to 43); and late fall, Nov (weeks 44 to 47)), as well as their two-way and three-way interactions. The main effects remained in all models, but non-significant interactions were removed from final models. Other variables were classified for inclusion in the models for evaluating effects on fertility such as lactation length (≤ 17 and ≥ 18 d), number of pigs weaned (≤ 7, 8 to 14, and ≥ 15), day of weaning (Monday and Thursday), and semen age (≤ 3 or 4 to 5 d) and were removed from final models if not significant. Sows in estrus on days 1 to 2 or not detected in proestrus or estrus by Day 6 after weaning and those with health issues were not included in the experiment. Certain measures that were unique to OvuGel such as administration at proestrus or estrus on Days 3, 4, or 5 after weaning were also tested. The Control group was also evaluated individually to determine effects of wean to estrus interval (Days 3 to 6) and individual inseminations (1^st^ or 2^nd^). The effects of ultrasound on fertility for OvuGel and Control treatments were evaluated for Thursday sows that were scanned and not scanned within the same week. Analysis of follicle data from the subpopulation that were scanned by ultrasound included the response variables of total number of follicles, follicle size, the proportion of females ovulating by Day 6, ovulation hour after weaning, inseminations occurring within the optimal time before ovulation (24 h before to 4 h after ovulation) and interval from insemination to ovulation. Continuous response measures were analyzed using the PROC MIXED procedure for significance of the main effects using the F-test and differences between least squares means identified using the t-test and the Tukey *posthoc* test for multiple comparisons when the main effect interactions were significant. Binary response measures were analyzed with PROC GLIMMIX using a binary distribution and a logit link function. Results are presented as Least Square Means ± SEM and differences between means were identified using the x^2^ test. Correlation and partial correlation coefficients for TRT were generated using the Proc Corr procedure in SAS using Pearson coefficients with continuous variables and Spearman when a binary variable was included. Significance was identified as *P* ≤ 0.05 and trends as *P* > 0.05 and ≤ 0.10.

## RESULTS

### Ovarian Measures in the Subpopulation of Sows After Weaning

In the sows scanned by transrectal ultrasound, no significant three-way interactions of the main effects were detected for any measure. The number of large follicles on Day 4 did not differ by TRT (17.5 ± 0.6), however inconsistent effects of parity were noted (**Table 1**). There was an interaction of TRT and season, with effects most clear with reduced numbers of follicles for sows weaned in Sep-Oct in OvuGel group (**Table 2**). Average follicle size on Day 4 did not differ by TRT (7.0 ± 0.1 mm) but was affected by parity (*P* < 0.0001), with P1 and P2 sows having smaller sized follicles than P3-6 and P7 sows (Table 1). The selection of sows eligible for OvuGel treatment based on detection of proestrus or estrus on days 3 to 5 associated with 90% of sows having large follicles on Day 4 and was similar to Controls (87%) that expressed estrus on days 3 to 6. Season effects also showed that sows weaned in Sep-Oct also had smaller ovulatory sized follicles than those weaned in the other seasons (Table 2). The proportion of sows ovulating within 6 days after weaning was affected by TRT with OvuGel increasing sows ovulating (92.2%) compared to Controls (79.4%). Further, sows with large follicles on Day 4 were more likely to ovulate (87.8%, *P* < 0.0001) by Day 6 than sows with medium size (62.1%). However, partial correlation indicated that follicle size on Day 4 was related with number of large follicles (r = -0.24) for both treatments but ovulation by Day 6 (r = 0.29) was only related with follicle size on Day 4 in the Control (r = 0.28, *P* < 0.0001), but not for OvuGel (r = 0.15, *P *> 0.05). Although there was no interaction with parity, a lower occurrence of ovulation was clearly evident in the Control for parities 1, 2 and ≥ 7 compared to OvuGel. The weaning to ovulation interval tended to be shorter in the OvuGel than Control, and was most related to the interaction of parity, where P1 in Control sows displayed a longer interval than P1 OvuGel sows (Table 1). The weaning to ovulation interval was influenced by season, with sows weaned in Sept-Oct having a shorter interval than those weaned in the other seasons. Inseminations occurring within the optimal time interval did not differ between OvuGel (86.8%) and Control (85.5%) or by parity but did show an interaction of TRT with season, with a lower proportion of Control sows receiving an optimal AI in Sep-Oct compared to OvuGel sows (Table 2). Analysis of follicle size and AI timing based on ultrasound in the sub-population indicated effects on FR were not significant, but numerical advantages for both TRT were in favor of large follicles (+ 10.9%) and an optimal AI interval (+ 6.2%).

**Table 1.** Least square means for reproductive response measures using ovarian ultrasound scanning for the subpopulation of weaned sows (n = 445) assigned to OvuGel or Control treatment (TRT) from different parities (P1, P2, P3 to 6, and ≥ 7)

	Treatment											
	Control				OvuGel							
	Parity											
	1	2	3 to 6	≥ 7	1	2	3 to 6	≥ 7	SE	*P*-value		
n	48	43	83	45	49	41	84	52		TRT^3^	Parity	TRT × Parity
Number of large follicles,^1^	15.4^b^	17.3^b^	17.8^ab^	19.6^a^	18.0^b^	17.0^b^	18.1^ab^	20.1^a^	1.1	ns	<0.05	ns
Follicle size, mm^1^	6.7^c^	6.9^b^	7.2^a^	7.3^a^	6.7^c^	7.0^b^	7.1^a^	7.3^a^	0.1	ns	<0.0001	ns
Ovulation within 6 d post weaning, %	63.3	78.7	89.2	80.7	91.7	93.0	93.1	92.4	4.2	0.004	0.04	ns
Weaning to ovulation interval, h	147.4	139.7	138.9	138.9	139.2	142.2	139.1	138.5	2.0	ns	0.1	0.1
Insemination within optimal time, %^2^	87.0	83.3	88.8	76.6	93.5	79.6	82.9	91.4	5.5	ns	ns	ns

^1^Data from the ultrasound on day four post-weaning. Large follicles from both ovaries were classified when ≥ 6.5 mm.

^2^Optimal time was considered within 24 h before ovulation and a maximum of 4 h post-ovulation.

^3^TRT = treatment.

^a-d^Within a row, means with different superscripts differ *P* ≤ 0.05.

ns = not significant.

**Table 2.** Least square means for reproductive response measures using ovarian ultrasound scanning for the subpopulation of weaned sows (n = 445) assigned to OvuGel or Control in different seasons represented by months (May-Jun, Jul-Aug, and Sep-Oct)

	Treatment									
	Control			OvuGel						
	Season									
	May-Jun	Jul-Aug	Sep-Oct	May-Jun	Jul-Aug	Sep-Oct	SE	*P*-value		
n	66	82	71	68	84	74		TRT^3^	Season	TRT × Season
Number of large follicles^1^	19.3^ab^	17.0^bcd^	16.3^cd^	18.2^bc^	20.8^a^	15.7^d^	0.9	ns	0.0008	0.02
Follicle size, mm^1^	7.2^a^	7.0^a^	6.9^b^	7.1^a^	7.1^a^	6.9^b^	0.1	ns	0.02	ns
Ovulation within 6 d post weaning, %	77.9	76.9	83.0	93.9	88.8	94.0	4.2	0.0004	ns	ns
Weaning to ovulation interval, h	142.0^a^	143.2^a^	138.5^b^	141.4^a^	141.3^a^	136.4^b^	1.4	ns	<0.01	ns
Insemination within optimal time, %^2^	89.1^ab^	89.1^ab^	75.7^c^	86.1^ab^	82.3^ab^	90.9^a^	5.5	ns	ns	<0.05

^1^Data from the ultrasound on day four post-weaning. Large follicles were classified when ≥ 6.5 mm.

^2^Optimal time was considered within 24 h before ovulation and a maximum of 4 h post-ovulation.

^3^TRT = treatment.

^a-d^Within a row, means with different superscripts differ *P* ≤ 0.05.

ns = not significant.

### Effect of Alternative OvuGel Administration Methods on Reproductive Responses

Analysis of ovarian follicle measures and responses for the subpopulation of sows scanned by ultrasound indicated significant effects in response to the day of application of OvuGel, but no interaction with parity or season (*P *> 0.05). Notably, the proportion of sows inseminated within the optimal time period was reduced when OvuGel was given on Day 3 compared to Days 4 and 5 (**Table 3**). But sows that received OvuGel on Day 5 were also less likely to ovulate by Day 6 (*P* < 0.0001) compared to OvuGel treatment on Days 3 or 4. For OvuGel treated sows, FR was lower for sows treated on Days 3 and 5 and pigs born alive was reduced for sows treated on Day 5 (Table 3). Measures for ovulation and Optimal AI timing as well as farrowing rate and litter sizes for OvuGel given based on signs of proestrus or estrus on Days 3 to 5 were not significant, although outcomes have limited meaning since the number of observations were highly unbalanced for day of OvuGel treatment.

**Table 3.** Least square means for the proportions of sows that received the single AI within the optimal time, that ovulated within six days, and the farrowing rate, total pigs born and born alive relative to the day of OvuGel treatment (day 3, 4, or 5) at proestrus or estrus after weaning and then followed 24 h later by a SFTAI

	OvuGel			
	Days			
	3	4	5	*P*-value
n¹	16	168	38	
Optimal AI time, %	57.1 ± 13.2^x^	85.7 ± 2.7^y^	100 ± 0.01^y^	0.03
Ovulation, %	93.8 ± 6.0^x^	95.8 ± 1.2^x^	66.7 ± 8.1^y^	< 0.0001
n²	135	1261	240	
Farrowing rate, %	76.2 ± 4.0^x^	87.0 ± 1.0^y^	80.0 ± 2.7^x^	0.0003
Total born	13.4 ± 0.4	13.3 ± 0.2	12.8 ± 0.3	ns
Born alive	11.9 ± 0.4^x^	12.2 ± 0.2^x^	11.4 ± 0.3^y^	0.02

¹Indicates the number of observations using ultrasound.

²Indicates the number of observations of inseminated sows.

^xy^Within a row, means with different superscripts differ *P* ≤ 0.05.

ns = not significant.

But there was an effect of day of weaning (P < 0.05) with the Monday wean having greater FR (*P* = 0.04) than the Thursday wean group (83.7 vs. 79.2 ± 2.0%). For the most fertile group with sows treated with OvuGel only on Day 4, FR (88.6 vs. 84.8 ± 1.4%), total born (13.5 vs. 13.1 ± 0.3) and born alive were all greater for the Monday compared to the Thursday wean groups (*P* < 0.05). Ultrasound scanning of the ovaries for Thursday weaned sows (n = 183) compared to those not scanned within the same week (n = 129) did not reduce farrowing rate or total born. However, semen age for the SFTAI was notably different between Thursday (4 to 5 d) and Monday (≤ 3 d) wean days due to day of semen collection and use and had a significant effect on farrowing rate (*P* < 0.05) but not litter size.

### Factors Affecting Reproductive Responses of Controls

Follicle size on Day 4, was negatively correlated (r = -0.22, *P* < 0.005) with wean to estrus interval (WEI). Optimal insemination timing for Controls based on estrus was not influenced by the WEI which averaged 4.7 ± 0.1 d. There was a significant effect of parity on WEI with P1 sows delayed (5.2 ± 0.1 d) compared to more mature sows (4.6 ± 0.1 d). For sows detected in estrus on Day 4, the first AI was less likely to occur within 24 h of ovulation when compared to those in estrus on Days 5 and 6 (**Table 4a**). The proportion of Control sows ovulating by Day 6 was greatest for those that expressed estrus on Day 4 compared to days 5 and 6. A total of 84% of the Control sows received a second insemination if detected in estrus the following day, and inseminations on Day 5 were most likely to be within the optimal interval relative to ovulation than the later days (**Table 4b**). Farrowing rate and litter traits were not affected by the day of insemination but was reduced in Control sows detected in estrus on only one day and receiving a single AI. Farrowing rate and litter traits were not affected by the WEI, or the day of weaning during the week (Monday or Thursday), or by semen age of the first or second insemination. Fertility measures were not affected in Control sows scanned by ultrasound every 8 h (n = 179) compared to those not scanned (n = 134) within the same weeks.

**Table 4.** Least square means for the proportions of Control sows that ovulated within the optimal time, ovulated within six days, the farrowing rate, total pigs born and born alive relative to the day of estrus and 1^st^ (a.) or 2^nd^ (b.) artificial insemination after weaning

a)					
	1st insemination				
	Days				
	3	4	5	6	*P*-value
n¹	3	85	74	7	
Optimal AI time %	66.7 ± 2.7	72.9 ± 4.8^x^	94.6 ± 2.6^y^	100 ± 0.1	0.01
Ovulation %	100.0 ± 0.1	95.5 ± 2.2^x^	80.4 ± 4.1^y^	25 ± 8.2^z^	< 0.0001
n²	54	845	624	153	
Farrowing rate %	92.2 ± 4.3	88.8 ± 1.2	86.3 ± 1.5	83.2 ± 3.0	ns
Total Born	13.4 ± 0.5	13.6 ± 0.2	13.9 ± 0.2	13.9 ± 0.3	ns
Born Alive	12.5 ± 0.5	12.3 ± 0.2	12.6 ± 0.2	12.2 ± 0.4	ns
b)					
	2nd insemination				
	Days				
	4	5	6	7	*P*-value
n¹	2	74	54	2	
Optimal AI time %	100.0 ± 0.1	81.1 ± 4.6	100 ± 0.1	100 ± 0.1	ns
n²	52	741	508	104	
Farrowing rate %	91.9 ± 4.5	89.8 ± 1.3	87.9 ± 1.5	82.9 ± 3.7	ns
Total Born	13.5 ± 0.5	13.6 ± 0.2	14 ± 0.2	13.7 ± 0.4	ns
Born Alive	12.5 ± 0.5	12.5 ± 0.2	12.7 ± 0.2	12.2 ± 0.4	ns

¹Indicates the number of observations using ultrasounds.

²Indicates the number of observations of inseminated sows.

^x-z^Indicates means are different within row (P ≤ 0.05).

ns = not significant.

### Comparison of OvuGel and Control Treated Sows for Fertility Responses

Because the alternative treatment Days 3 and 5 reduced fertility, we eliminated these and included only OvuGel treatment on Day 4 for comparison with Controls. There were no significant three-way interactions of the main effects, and no effect of TRT on FR or total born or born alive (**Table 5a**). The effect of day of weaning was still evident but did not interact with TRT. There was a trend for FR to be reduced and a significant reduction in total born for the Thursday compared to the Monday weaned sows (Table 5a). Because semen age differed by day of weaning, we replaced wean day with semen age as an explanatory variable and a significant effect on farrowing rate and a trend for a TRT × semen age interaction was evident. Farrowing rate reduction was most evident for the OvuGel treated sows that received a single insemination with semen 4 to 5 d old for the Thursday wean sows compared to the Monday weaned sows that were inseminated with semen ≤ 3 d old (**Table 5b**). Interactions of TRT with parity and season were significant (Table 4a). The interaction of TRT with parity was primarily due to OvuGel improving FR in P1 sows compared to Controls (**Table 6a**). The TRT interaction with season appeared to result from OvuGel increasing FR in Jul-Aug compared to Control (**Table 6b**), but both TRT showed the lowest FR in May-Jun, but without much effect in Sep-Oct and Nov.

**Table 5.** Least square means for reproductive fertility responses in response to treatment and Day of weaning (a.) and Days of semen storage age (b.)

a).									
		Treatment		Wean Day					
	n	Control	OvuGel^2^	Monday	Thursday	TRT^1^	Wean day	TRT × Parity	TRT × Season
Farrowing rate, %	2937	87.4 ± 1.0	86.7 ± 1.0	88.4 ± 1.0	86.1 ± 1.0	ns	0.07	< 0.005	0.05
Total Born	2549	13.4 ± 0.2	13.2 ± 0.2	13.4 ± 0.2	13.1 ± 0.2	ns	0.04	ns	ns
Born Alive	2549	12.3 ± 0.2	12.2 ± 0.2	12.4 ± 0.3	12.2 ± 0.2	ns	ns	ns	ns
b).									
		Treatment							
		Control			OvuGel				
		Semen Age							
	n	≤ 3 d		4-5 d	≤ 3 d	4-5 d		Semen age	TRT × Semen age
Farrowing rate, %	2806	88.2 ± 1.0		87.0 ± 1.5	91.3 ± 1.6	84.5 ± 1.5		0.02	0.06
Total Born	2441	13.4 ± 1.2		13.1 ± 0.2	13.4 ± 1.2	13.1 ± 0.2		ns	ns
Born Alive	2441	12.4 ± 0.3		12.2 ± 0.2	12.4 ± 0.3	12.2 ± 0.2		ns	ns

^1^TRT = treatment.

^2^OvuGel includes administration only on Day 4.

**Table 6.** Least square means for reproductive fertility responses by parity (a.) and season (b.) in response to OvuGel treatment only on Day 4 to Control

a)											
	Treatment										
	Control				OvuGel						
	Parity										
	1	2	3 to 6	≥ 7	1	2	3 to 6	≥ 7	SE	*P*-value	
n	244	399	573	460	154	302	447	375		TRT^1^	TRT × parity
Farrowing Rate %	76.5^d^	85.8^bc^	93.1^a^	89.4^ab^	87.9^ab^	83.4^cd^	89.0^ab^	87.9^bc^	2.0	ns	0.01
Total Born	13.9	14.3	13.4	11.9	13.6	13.8	13.1	12.0	0.03	ns	ns
Born Alive	13.0	13.3	12.4	10.6	12.7	13.0	12.1	10.7	0.3	ns	ns
b)											
	Treatment										
	Control				OvuGel						
	Season										
	May-Jun	Jul-Aug	Sep-Oct	Nov	May-Jun	Jul-Aug	Sep-Oct	Nov	SE	*P*-value	
n	410	561	467	238	325	392	355	206		TRT	TRT × season
Farrowing Rate %	79.6^c^	83.4^bc^	92.1^a^	90.7^a^	78.7^c^	89.2^a^	88.1^ab^	90.5^a^	2.0	ns	0.05
Total Born	13.7	12.7	13.5	13.5	13.3	12.8	13.0	13.4	0.3	ns	ns
Born Alive	12.4	11.9	12.4	12.6	12.2	11.9	12.2	12.3	0.3	ns	ns

^1^TRT = treatment.

^a-d^Within a row, means with different superscripts differ (P ≤ 0.05).

ns = not significant.

## DISCUSSION

This study was performed to evaluate whether ovulation induction using OvuGel followed by a SFTAI was appropriate for use in all parities during the summer and fall seasons. This is relevant because P1 sows generally have several measures of reduced fertility when compared to more mature sows. In addition, P1 and even multiparous sows are more susceptible to infertility in summer and fall compared to other seasons (De Rensis et al., 2017). The interest in SFTAI technology is focused on increasing use of genetically superior sires while using half of the sperm to produce a litter compared to use of two inseminations with conventional breeding. The results of this study are notable because they show that use of OvuGel based on proestrus or estrus was able to select 90% of sows with large follicles while 96% would ovulate by Day 6 and 86% were inseminated within the optimal fertilization window. Compared to Controls selected for fertility based on estrus, all measures were dependent upon the day of estrus and the days of the 1^st^ and 2^nd^ insemination. When including only treatment with OvuGel on Day 4, farrowing rate, total born and born alive matched that of the Control. This is very promising as it indicates that this treatment has the potential for wider commercial application and can be used in all sow parities and seasons to facilitate use of a SFTAI to aid in accelerating rate of genetic improvement.

Our second objective for this experiment was to evaluate each treatment for unique factors impacting their fertility performance. The concept behind needing flexibility for administering OvuGel on proestrus and estrus on Days 3 to 5, was to ensure that only fertile sows were treated and would account for faster follicle development by Day 3 and slower development by Day 5, while still maintaining overall group ovulation synchrony. The idea of the alternative administration on proestrus and estrus as opposed to the product label for Day 4 after weaning was to determine whether this might overcome treating sows with limited follicle development (Ulguim et al., 2018). The symptoms for identifying sows in proestrus were reported and included an array of behavioral and physical symptoms (Dillard and Flowers, 2020), but this can also come with challenges as the indicators can vary among females and their stage of maturity (Sterning et al., 1998). In the present study, proestrus was more difficult for the farm to identify as only 20% of the OvuGel sows were treated at this stage. Nevertheless, it was positive for the treatment and helped choose a highly fertile group of sows. Experiments from Dillard and Flowers (2020) evaluated commercial application of OvuGel based on Day 4 or 5 after weaning, based on estrus on those same days, or based on proestrus on Days 3 to 5. They reported reduced fertility when treatment occurred solely based on Day after weaning, while fertility was similar to controls when based on estrus on those days, and with excellent fertility when based on proestrus. In our study, OvuGel administration at proestrus or estrus on Days 3 or 5 compared to Day 4 resulted in reduced FR and associated with a reduction in litter size. A previous study indicated sows treated with OvuGel at 72 h had a longer treatment to ovulation interval compared to induction at 84 h and 96 h after weaning and that farrowing rates were reduced when females were treated at 72 or 84 h compared to 96 h (Knox et al., 2014). In the present experiment, OvuGel treatment on Day 4 was best for advancing ovulation and resulted in a FR similar to that of Controls. A previous study also treating sows on Day 4, noted that 88% of sows ovulated within 24 to 48 h after OvuGel, and that optimal fertility resulted with correct AI timing and adequate numbers of sperm (Knox et al., 2017). It is important to note that although optimal AI timing was not significant, there was a numerical increase in farrowing rate when this occurred. And in the OvuGel treatment, although ovulation induction by Day 6 was 92%, optimal AI timing did not keep pace (87%) suggesting an opportunity for improvement.

Also as part of our second objective, an assessment of factors affecting fertility performance in the Control was important to help us understand what natural sources of variation influenced follicle development, ovulation and AI timing. Our results match numerous studies showing a relationship between follicle development, estrus, and ovulation, and the factors that impact these measures (Kemp and Soede, 1996; Knox and Rodriguez-Zas, 2001; Lucy et al., 2001). Because Control sows are inseminated on each day detected in estrus, optimal AI timing can arise from the 1^st^ or 2^nd^ insemination based on the wean to estrus interval. In our study, not all Controls received an Optimal AI, but its’ importance to fertility is unclear since the 1^st^ or 2^nd^ second insemination could have sired some proportion of the pigs in the litter (McNamara and Knox, 2013; Ringwelski et al., 2013). But 16% of Control sows received only a single insemination and this did reduce fertility as has been reported elsewhere (Lamberson and Safranski, 2000). In our study, the wean to estrus interval did not affect farrowing rate and litter size, although this has been reported previously (Steverink et al., 1999) and shown from commercial industry records.

It was important to evaluate whether P1 sows should be used in an induction protocol during summer and fall. This is relevant as primiparous sows comprise ~20% of the sow herd, and are at greater risk for failures in estrus, ovulation and farrowing (Soede et al., 2012). This occurs due to several factors, but is thought to primarily involve their limitations for feed intake, smaller body size, and body reserves available to maintain body condition and normal metabolism during lactation (Iida and Koketsu, 2014; De Rensis et al., 2017; Koketsu et al., 2017). Any differences in follicle number or size measured on Day 4 would not be expected to result in response to treatment. Although we balanced sows by multiple criteria, there is the possibility that differences detected or not could arise from sow factors we did not measure or from variations in our ultrasound measures of follicles. Nevertheless, we did identify parity effects on follicle size in P1 and P2 sows. Smaller follicle size in primiparous sows has also been previously observed (Ulguim et al., 2018; Lopes et al., 2020) and these sows ovulate at a lower proportion in response to OvuGel (Knox et al., 2017). Lower fertility is also reported in P1 sows induced to ovulate with GnRH or other agonists when compared to multiparous sows. What was quite interesting in this experiment was that in P1 sows, OvuGel increased ovulation by Day 6 and this must be the factor that increased farrowing rate while matching litter size of Control, although the exact mechanism is not clear.

Seasonal decline in pig fertility is a common problem around the world and is most often associated with heat stress in summer but has also been linked to changes in photoperiod. The question for many farms that experience heat stress is whether ovulation induction and a single AI should be used during summer and into fall with animals that may have altered follicle development resulting from heat stress. Because the principle behind ovulation induction is based on control of the timing the LH surge and a synchronized ovulation among sows, the procedure has been of long-term interest for pig reproduction (Driancourt et al., 2013). If follicle development is delayed in summer and perhaps fall, then there could be advantages to delaying the timing of induction. Interestingly, detrimental effects were not apparent in summer but appeared in fall when the number and size of follicles were reduced. This corroborates with another study that found fewer large follicles in primiparous sows in early fall compared to late summer (Arend et al., 2019). However, other authors have reported fewer follicles at ovulation in both summer and fall compared to winter and spring (Lopes et al., 2014). Despite the similar decline in follicle development for both treatments in fall, OvuGel treatment increased ovulation by Day 6 in all seasons and must be the same factor that allowed the increase in farrowing rate in late summer. This effect should be explored, as this could be a key to improving the technology.

At the start of this study, we did not expect fertility to be affected by day of weaning. Our initial analyses included only the Thursday sows with data including the sub-population with follicle, ovulation and insemination measures. But a preliminary analysis using all of the data containing both wean days arrived at different fertility means than with the Thursday data set (Minton et al., 2019). Including wean day (Monday and Thursday) in the model result in the Thursday wean group having lower fertility than the Monday. We allotted sows from both wean days by the same criteria and accounted for most factors in our models. But throughout the study, data for age of semen was collected with particular interest for the SFTAI. An analysis showed that day of weaning was associated with differences in semen age at insemination, and when we tested that in the model, semen age had a significant effect on farrowing rate. For this farm, semen was collected and delivered to the sow farm on the same day but required 24 h until the semen was cleared for use. As it turned out, the Thursday weaned sows treated with OvuGel on Day 4 were all inseminated with semen 4 d old while the Monday OvuGel sows were inseminated with semen 1 d old. There was no effect on the Controls as most received two inseminations and only a single estrus day required that the 1^st^ AI was performed with semen 4 d old.

Early studies showed that sperm motility and acrosomes could change with storage, especially after 4 to 5 d which could link with fertilization and pregnancy rates (Waberski et al., 1994). There have been reports that sperm may or may not show decline in motility with storage depending upon the extender (De Ambrogi et al., 2006), and studies where no change in motility occurred with storage, but reduced fertility was evident with storage and extender (Kuster and Althouse, 1999). Most studies using modern long-term extenders have not observed changes in sperm quality for several days (Vyt et al., 2004; Estienne et al., 2007; Martín-Hidalgo et al., 2013) nor in breeding herd farrowing rates (Anil et al., 2004; Lucca et al., 2020). It is important to mention that most farms, regardless of use of long-term extender, do not use the semen past 3 d (Knox, 2016), and so outside of research studies or post-hoc analysis, many farms try to use semen by this day Our explanation for our observation that semen storage of 4 to 5 d reduced fertility might best be explained from data showing reduced fertility with a SFTAI with reduced numbers of sperm (Knox et al., 2017). Since the effect was only evident with OvuGel on Thursday with 4 to 5 d old semen, the lower fertility likely resulted from a reduction in the number of fertile sperm as the number of days of storage and handling increased. This could result from when doses are removed and returned when not used for breeding, and when the storage unit door is repeatedly opened and closed on commercial farms during the summer. If results differ from controlled experiments, it is important to remember that most published extender storage studies use double insemination protocols. So, while the long-term extender itself will have little impact, we suspect the semen handling for a SFTAI in summer would require more specific handling and storage instructions especially if doses would be used beyond 3 d.

## CONCLUSION

The results of this study show that use of OvuGel based on proestrus or estrus on Day 4 after weaning and followed by a SFTAI, could match the farrowing rate and litter size of sows bred conventionally during summer and fall. The proestrus and estrus approach was an advantage in selecting fertile sows for treatment. However, OvuGel given on Days 3 or 5 based on proestrus or estrus was detrimental to fertility. Despite P1 sows having smaller follicles, OvuGel increased ovulation by Day 6 and was the only factor most likely to be associated with the increase in farrowing rate. Similarly, season affected follicles, and as OvuGel was able to induce ovulation in all seasons, this was the likely factor that allowed the increase in farrowing rate in late summer. For sows assigned for ovulation induction and receiving only a single fixed time insemination, it will be important to monitor on-farm semen handling especially in hot seasons and when using semen doses beyond 3 d in storage. Overall, there appears to be exciting opportunities to use this technology for increasing the rate of gene transfer.
